# Dexamethasone vs methylprednisolone high dose for Covid-19 pneumonia

**DOI:** 10.1371/journal.pone.0252057

**Published:** 2021-05-25

**Authors:** Miguel Alejandro Pinzón, Santiago Ortiz, Héctor Holguín, Juan Felipe Betancur, Doris Cardona Arango, Henry Laniado, Carolina Arias Arias, Bernardo Muñoz, Julián Quiceno, Daniel Jaramillo, Zoraida Ramirez

**Affiliations:** 1 Department of Infectious Diseases, Clínica Medellín, Grupo Quirónsalud, Medellín, Antioquia, Colombia; 2 Department of Mathematical Sciences, Universidad EAFIT, Medellín, Antioquia, Colombia; 3 Pharmaceutical Service, Clínica Medellín, Grupo Quirónsalud, Medellín, Antioquia, Colombia; 4 Department of Internal Medicine, Clínica Medellín, Grupo Quirónsalud, Medellín, Antioquia, Colombia; 5 Department of Public Health, CES University, Medellín, Antioquia, Colombia; 6 Department of Epidemiology, CES University, Medellín, Antioquia, Colombia; 7 Department of Pulmonology, Clínica Medellín, Grupo Quirónsalud, Medellín, Antioquia, Colombia; 8 Department of Rheumatology and Autoimmune Diseases, CES University, Clínica León XIII, Medellín, Antioquia, Colombia; Kaohsuing Medical University Hospital, TAIWAN

## Abstract

**Background:**

There is no effective therapy for the severe acute respiratory syndrome by coronavirus 2 (SARS-CoV2) responsible for the Coronavirus disease 2019 (Covid-19). To date, dexamethasone has shown a decrease in mortality in patients who require oxygen, especially those with invasive mechanical ventilation. However, it is unknown if another corticosteroid can be used, the optimal dose and its duration, to achieve a better clinical outcome. The objective of the study was to compare the differences in clinical outcome and laboratory results in hospitalized patients with severe SARS-CoV2 Pneumonia treated with dexamethasone at 6 mg doses versus patients treated with high-dose methylprednisolone.

**Materials and methods:**

Ambispective cohort study with survival analysis of 216 patients diagnosed with severe Covid-19 pneumonia confirmed by polymerase chain reaction for SARS-CoV2 by Berlin protocol, who were hospitalized in a high-complexity clinic in Medellín, Colombia. The patients should also have supplementary oxygen and radiological confirmation of Pneumonia by chest tomography. Sample size was not calculated since the total population that met the inclusion criteria was evaluated. 111 patients were treated with the institutional protocol with intravenous dexamethasone 6 mg QD for seven to 10 days if they required oxygen. Since September 15, 2020, the hospitalization protocol of the clinic was modified by the Infectious Diseases and Pulmonology service, recommending a high dose of methylprednisolone of 250 to 500 mg every day for three days with a subsequent change to oral prednisone 50 mg every day for 14 days. The protocol was not applied in the intensive care unit, where dexamethasone continued to be administered. The clinical outcome and differences in laboratory results of the patients who received dexamethasone vs. the prospective cohort that received methylprednisolone from September 15 to October 31, 2020, were evaluated. Follow-up was carried out by outpatient consultation one month after discharge or by telephone, inquiring about readmission or living-dead status.

**Results:**

216 patients had Covid-19 pneumonia documented by ground-glass imaging and alveolar pressure / inspired oxygen fraction (PaFi) less than 300. 111 patients received dexamethasone (DXM) and 105 received methylprednisolone (MTP). Patients in the DXM group evolved to severe ARDS in a higher proportion (26.1% vs 17.1% than the MTP group). Upon completion 4 days of treatment with parenteral corticosteroid, laboratory markers of severity decreased significantly in the group that received MTP, CRP 2.85 (2.3–3.8) vs 7.2 (5.4–9.8), (p-value < 0.0001), D-dimer 691 (612–847) vs 1083 (740–1565) (p-value = 0.04) and DHL 273 (244–289) vs 355 (270.6–422) (p-value = 0.01). After starting the corticosteroid, transfer to the intensive care unit (4.8% vs. 14.4%) and mortality (9,5% vs. 17.1%) was lower in the group that received MTP. Recovery time was shorter in patients treated with MTP, three days (3–4) vs. DXM 6 days (5–8) (p-value < 0.0001). At 30-day follow-up, 88 (92.6%) were alive in MTP vs 58 (63.1%) of those who received dexamethasone.

**Conclusions:**

In this study, the treatment of severe Covid-19 Pneumonia with high-dose methylprednisolone for three days followed by oral prednisone for 14 days, compared with 6 mg dexamethasone for 7 to 10 days, statistically significantly decreased the recovery time, the need for transfer to intensive care and the severity markers C-reactive protein (CRP), D-dimer and LDH. Randomized controlled studies with methylprednisolone are required to corroborate its effect, and studies in a population hospitalized in intensive care wards.

## Introduction

Coronavirus disease 2019 (Covid-19) is caused by the virus called Severe Acute Respiratory Syndrome Coronavirus 2 (SARS-CoV2), an emerging pathogen initially identified in Wuhan, China in December 2019 [1]. Until November 11, 2020, 52,024,841 people are infected globally, with 1,282,944 deaths, with the lethality of 2.5% [2] with a greater extension than the previous epidemics by SARS-CoV and MERS [3].

To face the pandemic, drugs used in the previous SARS-COV and MERS epidemics, including chloroquine and hydroxychloroquine [4], lopinavir/ritonavir [5], azithromycin [6], and ivermectin [7], among others, showed some usefulness in vitro against SARS-CoV2. In a retrospective multicenter study in Michigan, United States, the administration of hydroxychloroquine alone or in combination with azithromycin was associated with a reduction in mortality [8]. However, in randomized clinical trials, no favorable effect was evidenced.

On June 16, 2020, the Recovery study’s preliminary report [9] was published, which compared patients with Covid-19 Pneumonia who received dexamethasone 6 mg per day for up to 10 days (or until hospital discharge) against those who did not receive corticosteroids. In this study, mortality was lower in the patients who received dexamethasone in comparison with the group that did not receive it. Although the decrease in mortality was 11% in patients with invasive mechanical ventilation (29% vs. 40%), the difference in global mortality was very narrow (22.9% vs. 25.7%), Given the results of RECOVERY Study, most of the hospital management guidelines worldwide included it. However, it is not clear so far if the treatment for the complications of Covid-19, Acute Respiratory Distress Syndrome (ARDS) [10] or Cytokine Release Syndrome (CRS) [11], is a particular effect of dexamethasone or is a class effect in which other corticosteroids can be used, or even if the low or high doses of corticosteroids are similar in this effect. The objective of the study was to compare the clinical outcome of patients treated with dexamethasone compared with high-dose methylprednisolone at the hospital level in a high-complexity clinic in Medellín, Colombia.

## Materials and methods

Ambispective cohort study with survival analysis was conducted in a high-complexity clinic in Medellín, Colombia.

The study included patients over 18 years of age, hospitalized with Covid-19 pneumonia confirmed by positive Real-Time Reverse Transcription Polymerase Chain Reaction for SARS-CoV2 (RT-PCR SARS-Cov2) by Berlin protocol. The patients should also have supplementary oxygen and radiological confirmation of Pneumonia by chest tomography. Sample size was not calculated since the total population that met the inclusion criteria was evaluated.

After obtaining informed consent, patients were treated according to the institutional protocol (from June 11 to September 14, 2020) with dexamethasone 6mg intravenous daily for up to 10 days (or until hospital discharge) if the patient required supplemental oxygen (retrospective cohort). Since September 15, 2020, the management protocol was changed from dexamethasone to methylprednisolone 250 to 500 mg daily for three days, followed by prednisone 50 mg orally every day for 14 days (prospective cohort). The median dose of methylprednisolone was 500mg intravenous day for three days. The dose was based on management reports in SARS-CoV and the experience of management of fulminant Pneumonia and organized Pneumonia in the institution. All patients received ivermectin one drop/kg for three days to prevent Loeffler syndrome due to corticosteroids (one drop equals 200 micrograms of ivermectin). All patients started treatment on the first day of hospitalization and were not randomly selected.

As exclusion criteria to enter any cohort of the study, contraindications associated with corticosteroids were considered, dissent for medical management, death in the first 24 hours, patient in palliative care or with a life expectancy of less than six months. If the patient required admission to the ICU and did not receive at least two doses of the corticosteroid, was withdrawn from the cohort to follow (In the ICU protocol, only dexamethasone 6 mg is given intravenously). If the patient receives at least two doses of methylprednisolone but did not continue with prednisone, they were not included, but their outcome continued to be monitored. Patients who also received less than two days of dexamethasone treatment were withdrawn from study follow-up. Colchicine was administered by clinic protocol since July 1; this variable was included in the patients evaluated.

Upon admission, laboratory tests were performed, such as hemogram, kidney, liver function tests, arterial blood gases, lactate dehydrogenase, D-dimer, serum ferritin, and C-reactive protein. Low molecular weight heparins were prescribed to all patients to prevent thromboembolism during their hospital stay.

Pneumonia was classified as severe by the presence of hypoxemia or the need for supplemental oxygen, and in some cases, complicated with septic shock syndrome, or multisystem compromise. Acute Respiratory Distress Syndrome (ARDS) was defined by the presence of bilateral pulmonary infiltrates not explained by an etiology other than Covid-19 and PaFi less than 300. The arterial blood gases results during each patient hospitalization were evaluated to determine the evolution of ARDS. The primary outcome is Recovery time, defined as significant clinical improvement in the evolution of the patient to consider discharge. Thus, subjective improvement of dyspnea was required, reduction of oxygen support at least until oxygen was available through nasal cannula (if previously on oxygen for ventury, non-rebreathing mask, non-invasive mechanical ventilation or invasive mechanical ventilation) or supplementary oxygen removal. Secondary outcomes are transfer to intensive care unit (ICU), mortality and readmission 30 days after discharge, hospital over infection. Follow-up was carried out by outpatient consultation one month after discharge or by telephone, inquiring about readmission or living-dead status.

Standardization was carried out in the observation of the researcher, thus guaranteeing adequate techniques in collecting information. With these data, a database was built in Microsoft Excel, and before the analysis, it was subjected to quality control.

Once all the variables had been collected, the quantitative variables were expressed in terms of their sample median (together with their respective 95% bootstrap confidence intervals) and were compared using a two-sided Mann & Whitney hypothesis test. Qualitative variables were expressed in absolute values, along with their respective percentage values (%) and were compared using a two-sample z-test for proportions hypothesis test. Comparing the methylprednisolone (MTP) vs. dexamethasone (DXM) treatment performance was carried out through the response variable ’Recovery Time’, measured in days, which expresses the recovery time until discharge if at least two doses of the respective treatment have been received. This analysis was carried out through a survival analysis model with Cox regression [12], and it was proved that the risks were proportional, from two stages: in the first, a robust Cox regression [13] was carried out to identify the predictor variables that explain the hazard ratio (HR), avoiding a possible interference of outlier observations in the partial likelihood estimation. In the second stage, with the significant variables (’Treatment’ and ’Colchicine’), a new model was estimated for the point estimates and their 95% confidence intervals. These statistical analysis were performed in the R statistical software [14] through the *coxrobust* package [15] to perform the robust Cox regression and *survival* package [16] for traditional Cox regression.

### Ethical considerations

The Clinica Medellin ethics committee approved the study. Informed consent was obtained from the study participants.

Trial registration number ISRCTN33037282.

## Result

**Fig 1:**
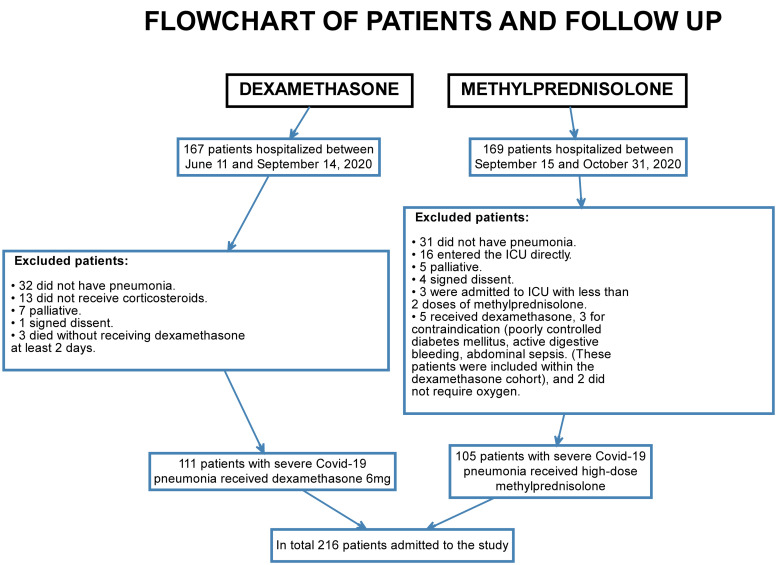
Flowchart of patients and follow up.

In total, 216 patients were included, 111 received dexamethasone (retrospective DXM cohort), and 105 patients received methylprednisolone (prospective MTP cohort) (Fig 1). There were no statistically significant differences between the two groups of patients, impairing comparability in the variables of age, sex, comorbidities, initial: PaFi, CRP, DHL, Ferritin, D-dimer, or use of antibiotics. Colchicine was prescribed to 100 patients (95.2%) of the MTP group vs. 88 (79.3%) of DXM (Table 1).

**Table 1:** Sociodemographic and clinical characteristics of hospitalized patients with Covid-19 pneumonia, according to treatment group.

Variable	Treatment		Mann & Whitney	Proportions Z-test	Total Patients (n = 216)
	MTP (n = 105)	DXM (n = 111)	P-Value	P-Value	
Age	64 (60–68)	63 (58–69)	0.94	--	64 (60–68)
Sex					
Male	67 (63.8%)	60 (54.1%)	--	0.19	127 (58.8%)
Female	38 (36.2%)	51 (45.9%)	--		89 (41.2%)
Comorbidities	77 (73.3%)	84 (75.7%)	--	0.81	161 (74.5%)
Cancer	6 (5.7%)	7 (6.3%)	--	1	13 (6%)
Hypothyroidism	7 (6.7%)	11 (9.9%)	--	0.54	18 (8.3%)
Heart Disease	10 (9.5%)	9 (8.1%)	--	0.89	19 (8.8%)
Renal Failure	7 (6.7%)	13 (11.7%)	--	0.29	20 (9.3%)
Dementia	6 (5.7%)	5 (4.5%)	--	0.92	11 (5.1%)
Obesity	12 (11.4%)	8 (7.2%)	--	0.4	20 (9.3%)
COPD	11 (10.5%)	12 (10.8%)	--	1	23 (10.6%)
Diabetes Mellitus	21 (20%)	31 (27.9%)	--	0.23	52 (24.1%)
Hypertension	49 (46.7%)	55 (49.5%)	--	0.77	104 (48.1%)
Initial ARDS					
Mild	79 (75.2%)	80 (72.1%)	--	0.71	159 (73.6%)
Moderate	17 (16.2%)	13 (11.7%)	--	0.45	30 (13.9%)
Severe	9 (8.6%)	15 (13.5%)	--	0.35	24 (11.1%)
Unknown	0 (0%)	3 (2.7%)	--	0.27	3 (1.4%)
Initial PaFi	253 (231–265.9)	266 (233–290)	0.59	--	255 (240–271)
Initial CRP	11.85 (9.7–15.9)	11.2 (9.7–12.9)	0.42	--	11.4 (10.1–12.9)
Initial LDH	299 (275–324)	334 (297.5–370.5)	0.23	--	309 (296–336)
Initial Ferritin	470.5 (364–726)	513.5 (395.5–764)	0.75	--	495 (404–672)
Initial D-Dimer	709 (577–849)	868 (689–1079)	0.24	--	774 (687–876)
Antibiotics	103 (98.1%)	100 (90.1%)	--	0.03	203 (93.9%)
Colchicine	100 (95.2%)	88 (79.3%)	--	0.001	188 (87%)

**COPD:** Chronic Obstructive Pulmonary Disease; **ARDS:** Acute Respiratory Distress Syndrome; **CRP:** C-reactive protein; **DXM:** Dexamethasone; **LDH:** Lactate Dehydrogenase; **PaFI:** arterial oxygen pressure / inspired oxygen fraction

### Clinical outcome

The patients in DXM group evolved to severe ARDS in a higher proportion (26.1% vs 17.1% in the MTP group). Upon completing 4 days of treatment with parenteral corticosteroid, the paraclinical markers of severity decreased significantly in the group that received MTP, with CRP 2.85 (95% CI: 2.3–3.8) vs 7.2 (5.4–9.8), (p-value < 0.0001), D-dimer 691 (95% CI: 612–847) vs 1083 (95% CI: 740–1565) (p-value = 0.04) and DHL 273 (95% CI: 244–289) vs 355 (95% CI: 270.6–422; p-value = 0.01).

Transfer to the intensive care unit and mortality after starting the corticosteroid was lower in the group that received MTP (4.8% vs. 14.4%) and (9.5% vs. 17.1%), respectively. (Table 2).

**Table 2:** Clinical outcome of hospitalized patients with Covid-19 pneumonia, according to the treatment group.

Variable	Treatment		Mann & Whitney	Proportions Z-test	Total Patients (n = 216)
	MTP (n = 105)	DXM (n = 111)	P-value	P-Value	
ARDS Evolution					
Mild	61 (58.1%)	61 (54.9%)	--	0.74	122 (56.5%)
Moderate	24 (22.9%)	21 (18.9%)	--	0.56	45 (20.8%)
Severe	18 (17.1%)	29 (26.1%)	--	0.15	47 (21.8%)
Lung Over Infection	2 (1.9%)	4 (3.6%)	--	0.73	6 (2.8%)
Hyperglycemia	8 (7.6%)	11 (9.9%)	--	0.72	19 (8.8%)
Invasive Mechanical Ventilation	3 (2.9%)	22 (19.8%)	--	0.0002	25 (11.6%)
Posterior PaFi	263.5 (250–285)	262 (246–287)	0.67	--	262 (250–282)
Posterior CRP	2.85 (2.3–3.8)	7.2 (5.4–9.8)	< 0.0001	--	4.3 (3.5–5.4)
Posterior LDH	273 (244–289)	355 (270.6–422)	0.01	--	281.5 (259–301)
Posterior Ferritin	476 (362–644)	580 (496.5–669)	0.20	--	537 (460–626)
Posterior D-Dimer	691 (612–847)	1083 (740–1565)	0.04	--	807 (642.3–1009)
ICU Admission After Corticosteroid Treatment	5 (4.8%)	16 (14.4%)	--	0.03	21 (9.7%)
Mortality	10 (9.5%)	19 (17.1%)	--	0.15	29 (13.4%)
Recovery Time	3 (3–4)	6 (5–8)	< 0.0001	--	4 (4–5)
Recovery time (with Colchicine)	3 (3–4)	5 (4–6.5)	0.003	--	4 (3–5)
30-day Status					
Alive	88 (92.6%)	58 (63.1%)	--	< 0.0001	146 (78.1%)
Death at Home	1 (1.5%)	5 (5.4%)	--	0.34	6 (3.2%)
Re-admitted and Alive	0 (0%)	4 (4.3%)	--	0.12	4 (2.1%)
Death Upon Re-admitted	1 (1.5%)	0 (0%)	--	1	1 (0.5%)
Unknown	5 (5.3%)	25 (27.2%)	--	0.0001	30 (16%)

**ARDS:** Acute Respiratory Distress Syndrome; **CRP:** C-reactive protein; **DXM:** Dexamethasone; **ICU:** Intensive Care Unit; **LDH:** Lactate Dehydrogenase; **PaFI:** arterial oxygen pressure / inspired oxygen fraction.

**Fig 2:**
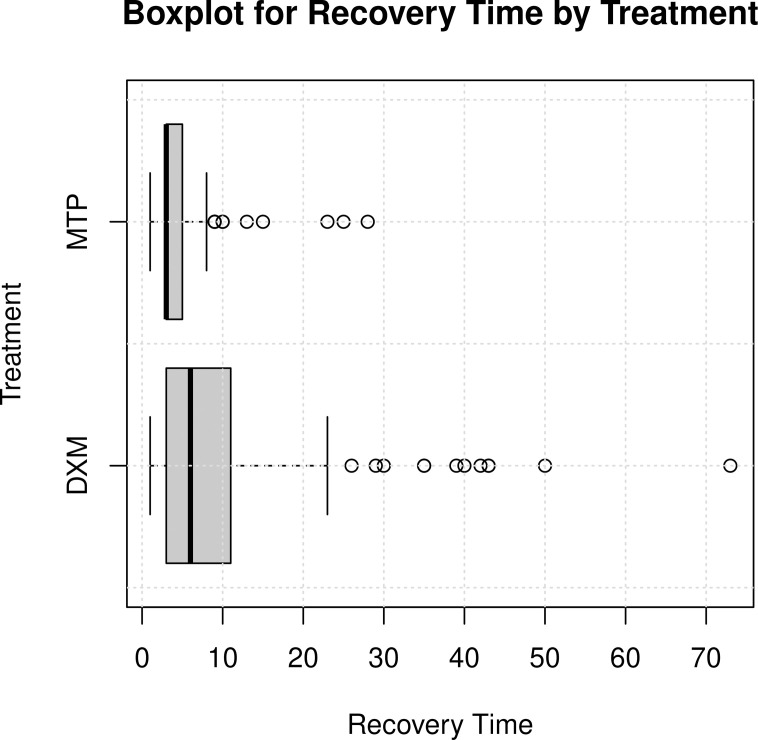
Recovery time according to the treatment received: Dexamethasone vs. methylprednisolone.

Recovery time was shorter in the patients treated with MTP, three days (3–4) vs. DXM 6 days (5–8) (p-value < 0.0001). (Fig 2). At 30-day follow-up, 88 (92.6%) were alive in MTP vs 58 (63.1%) of those who received dexamethasone.

### Recovery time performance analysis

Through a proportional hazards model, the impact of the variables on the hazard rate function of the response variable ’Recovery Time.’ From the robust Cox model was demonstrated, only the variables ’Treatment’ and ’Colchicine’ were significant for modeling the hazard rate function. A second Cox regression was performed with the first model’s significant variables to model the risk rate, also observing its significance (Table 3).

**Table 3:** Recovery time performance analysis—Cox models.

Explanatory Variable	Robust Cox Regression (Model 1)			Cox Regression (Model 2)		
	Coef.	Exp(Coef)	P-value	Coef.	Exp(Coef)	P-value
Treatment (1 –MTP and 0 –DXM)	0.97	2.63	< 0.0001	0.57	1.76 (1.29–2.40)	0.0003
Colchicine (1 –Received and 0 –Not received)	0.87	2.39	0.025	0.89	2.44 (1.45–4.09)	0.0007
Age	-0.01	0.99	0.26	--	--	--
Sex (1 –Male and 0 –Female)	-0.19	0.82	0.29	--	--	--
Comorbidities (1 –Yes and 0 –No)	0.33	1.40	0.15	--	--	--

In this study, the survival function $\hat{S}$ indicates the estimated *probability that a patient required more days to recover*, and the hazard rate function $\hat{\lambda }\left(t\right)$ the probability intensity that a patient is recovering at an instant of time *t* + *Δ* since, at time *t* it has not recovered, with *Δ* > 0.

**Fig 3:**
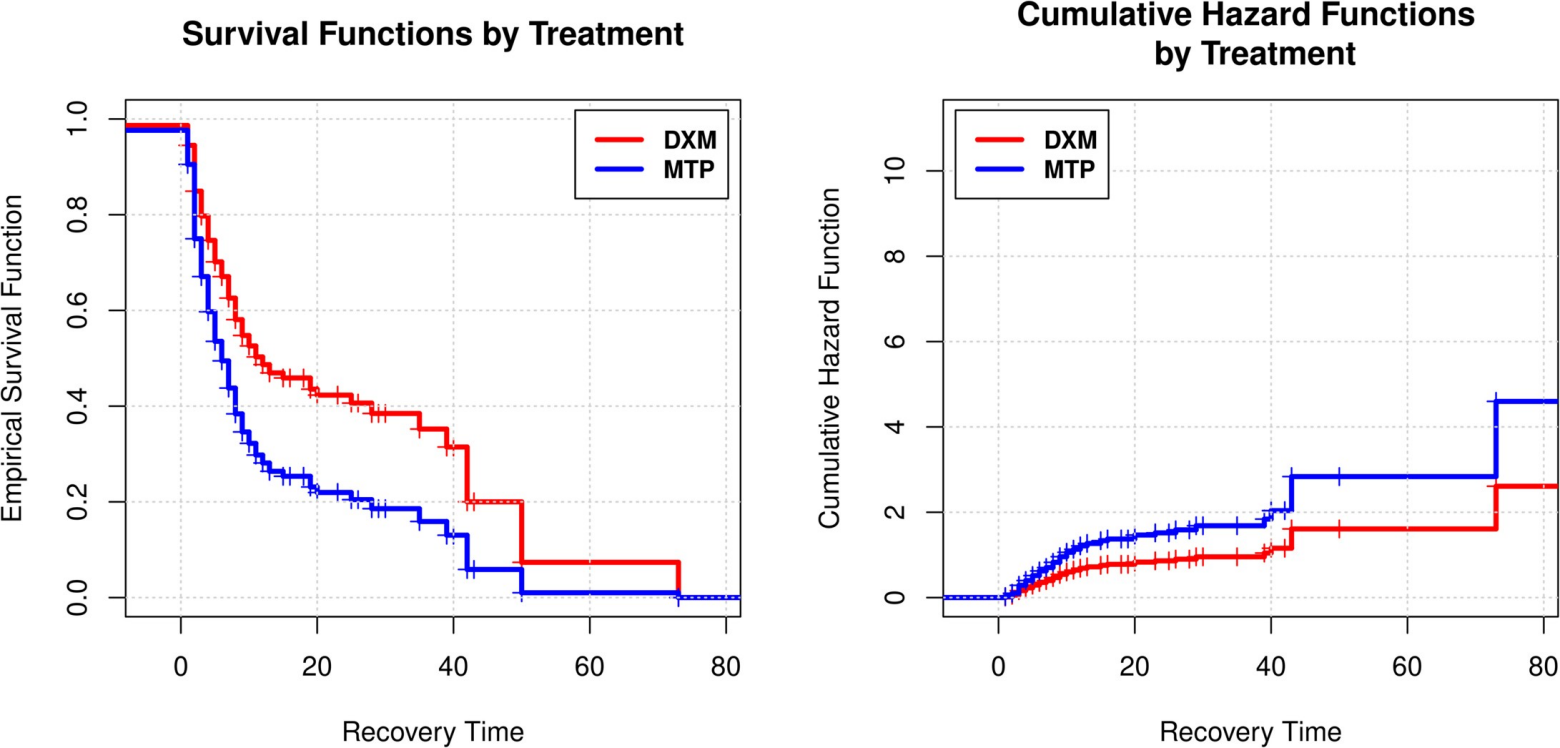
**A.** Survival function estimated by Cox model, according to treatment received: MTP vs DXM. **B.** Cumulative Hazard function estimated by Cox model, according to the MTP vs DXM treatment.

For the variable ’Treatment,’ the estimated regression coefficient of +0.57 indicated that the treatment with methylprednisolone had a positive impact with a value of 1.76 (1.29–2.40) in the hazard rate function $\hat{\lambda }\left(t\right)$, that is, patients who received methylprednisolone had a greater chance of instant recovery from Covid-19 compared to patients who received DXM (Fig 3A). Patients who received MTP had a higher cumulative risk of recovering in an instant of time *t* + *Δ* since at time t has not recovered (Fig 3B).

**Fig 4:**
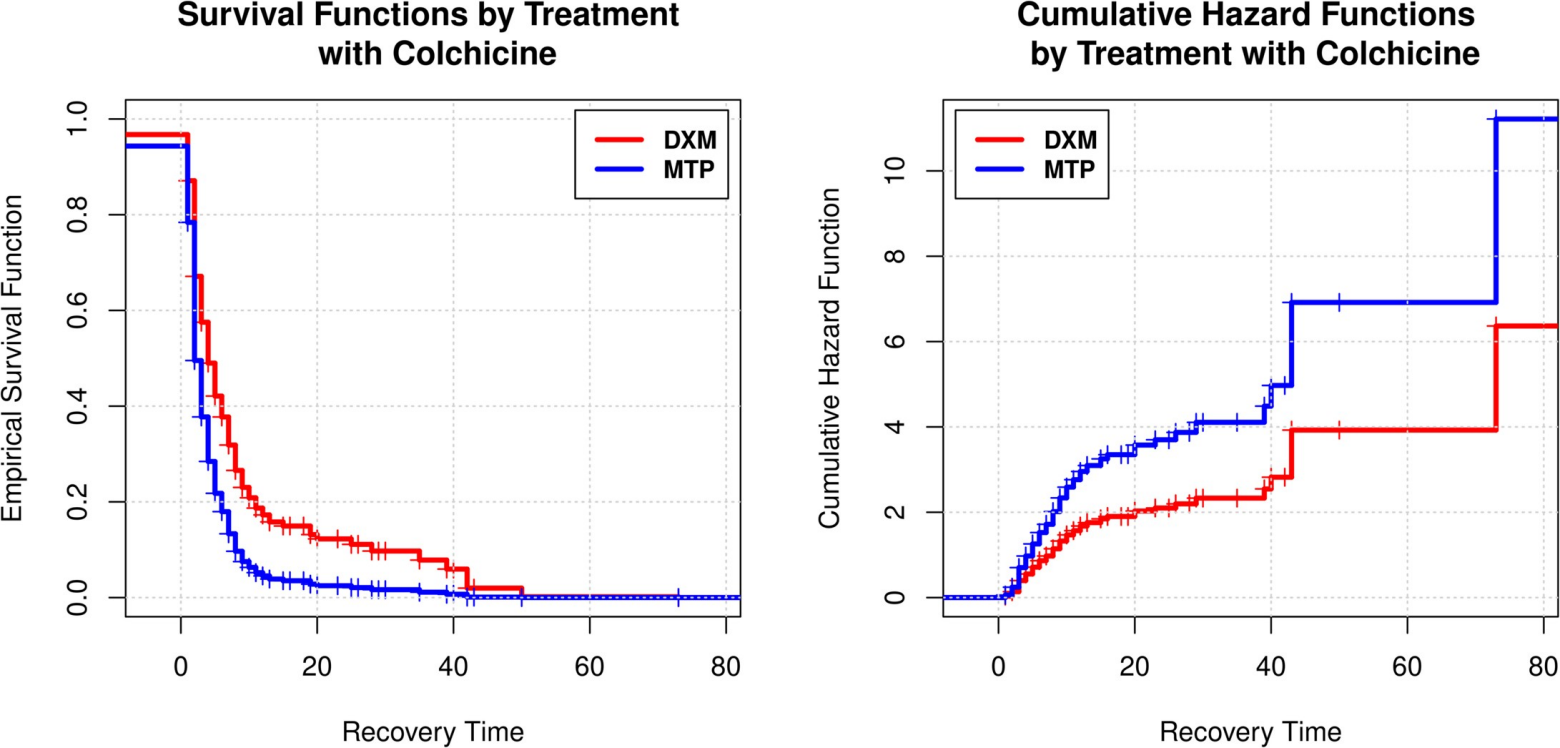
**A**. Survival function estimated by the Cox model, according to whether they received corticosteroid with colchicine. **B.** Cumulative hazard function estimated by the Cox model if the drug colchicine was added to the corticosteroid.

The same happened with the variable ‘Colchicine’; for this, an estimated regression coefficient of +0.89 was obtained, indicating that the application of Colchicine had a positive impact with a value of 2.44 (1.45–4.09) in the hazard rate function $\hat{\lambda }\left(t\right)$, that is, patients who received Colchicine had a greater chance of instant recovery from Covid-19 compared to those who did not (Fig 4A). Patients who received Colchicine added to the corticosteroid had a greater cumulative risk of recovering in an instant of time *t* + *Δ* given that at time t they had not recovered (Fig 4B).

## Discussion

To date, no medications have shown efficacy against SARS-COV2. Remdesivir, although approved by the Food and Drug Administration (FDA) in the United States as an antiviral for SARS-COV2, has been questioned due to its limited clinical effect [17], The current focus has been directed towards the complications associated with Covid-19: ARDS and Cytokine Release Syndrome, both characterized by an increase in tumor necrosis factor-alpha (TNF alpha), interleukin (IL) 1B, IL-2 IL- 6, IL-8, IL-10, and interferon y (IFN y) [18] that generate a dysregulated autoinflammatory response, severe tissue and systemic inflammation, and eventually death. Therefore, corticosteroids have been used, drugs with powerful anti-inflammatory action [19]. As a historical background, in the SARS epidemic in Guangzhou, China, which occurred in 2003, the infection was associated with a clinical picture similar to the current disease Covid-19, Zhao et al. [20], Compared four types of treatment for patients with SARS-CoV pneumonia, which included different antibiotics, antivirals, and in some, the administration of corticosteroids at different doses. Only the patients who received methylprednisolone in high doses of 160-1000mg per day for 5 to 14 days did not require mechanical ventilation, without presenting mortality.

At the beginning of the Covid-19 pandemic, the administration of corticosteroids was controversial [21]. However, recent evidence changed ARDS ICU management secondary to other etiologies. Villar et al. [22], in a placebo-controlled, randomized, and multicenter study, found that patients with ARDS treated with dexamethasone had lower mortality (21% vs. 36%, p-value < 0.0047). Wu et al [23]. described a group of patients with ARDS secondary to Covid-19, treated with methylprednisolone, with a lower risk of death (HR, 0.38; 95% CI, 0.20–0.72). Recently, the RECOVERY results modified the treatment guidelines [9]. In this study, patients who received dexamethasone had a decrease in mortality in a third of ventilated patients and in a fifth in other patients who received oxygen only. However, the difference in all patients mortality was 22.9% in those who received dexamethasone 6 mg vs. 25.7% in those who did not receive it.

In Covid-19 patients, the high mortality can be explained by the rapid development of Organized Pneumonia secondary to SARS-CoV2, since its appearance even in the first week of infection has been documented in autopsies. This pathology generally requires treatment with high doses of corticosteroids, cited by some as "pulse" doses and longer duration. Therefore, the dose suggested by RECOVERY could be insufficient for a high percentage of patients [24].

Yang et al. [25], evaluated 175 patients with severe Covid-19, documenting in the multivariate analysis as a protective factor against progression to critical disease the administration of methylprednisolone (p-value <0 .001; OR: 0.054 95% CI: 0.017–0.173).

Edalatifard et al. [26], In a small clinical trial, randomized 34 patients with Covid-19 pneumonia to receive methylprednisolone 250 mg per day for three days vs. 34 patients managed with standard care. Patients with clinical improvement were higher in the methylprednisolone group than in the standard care group (94.1% versus 57.1%), and the mortality rate was lower in the methylprednisolone group (5.9% versus 42.9%; p-value < 0.001).

Ruiz-Irastorza et al. [27], In a comparative observational study of patients with Covid-19 pneumonia, compared patients who received week-2-MTP (125–250 mg / d for three days) with those who did not. The adjusted HRs for death and death or intubation for patients in the week-2-MTP group were 0.35 (95% CI 0.11 to 1.06, p-value = 0.064) and 0.33 (95% CI 0.13 to 0.84, p-value = 0.020), respectively.

This study evaluated the differences between clinical and laboratory outcomes in patients treated with high-dose dexamethasone or methylprednisolone. We showed lower mortality in the methylprednisolone group and a shorter recovery time of the patients, a finding that had not been reported so far. The explanation seems to correspond to a dose-dependent effect of the corticosteroid, which observed a more significant decrease in the inflammatory response in this group of patients than dexamethasone, decreasing CRP, LDH, and D-dimer. These laboratories, which have been proposed as markers of severity in Covid-19, were not available at baseline or follow-up as a response after therapy in the RECOVERY study. Despite receiving high doses of corticosteroids, there was no increased risk of superinfection, possibly due to the short time of administration. Two over infections (1.9%) vs. DXM 4 (3.7%) occurred in the MTP group, corresponding in all cases to ventilator-associated pneumonia. Hyperglycemia was observed in some diabetic patients in both treatment groups.

Patients in the DXM group evolved to severe ARDS in a higher proportion than the MTP group, and transfer to the intensive care unit after starting the corticosteroid was less in the group that received MTP which is related to less clinical deterioration and less progression to critical illness with the administration of high dose methylprednisolone. Likewise, the recovery time was shorter in patients treated with MTP, whose connotation more important during the current Covid-19 pandemic, is that it can favor an earlier discharge from the hospital and thus avoid the collapse of the hospital system.

The presented medical protocol included the use of colchicine 0.5mg every 12 hours, for up to 14 days since July 1, 2020. In order to avoid bias in corticosteroids performance results, our main objective, we assessed the use of colchicine as a secondary finding in the research. The use of colchicine is supported by other recent studies. In the GRECCO randomized clinical study [28], the use of colchicine decreased the primary endpoint of time to clinical deterioration in 1.8% (1 of 55 patients) vs 14.0% in the control group (7 of 50 patients), with odds ratio value 0.11, 95% CI (0.01–0.96) and significant p-value  =  0.02. Moreover, in Scarsi et al., [29], colchicine is tested in patients with Covid-19 pneumonia and ARDS, where it shows better survival rate compared with standard-of-care at 21 days of follow-up (84.2% vs 63.6%, p-value = 0.001).

As the main strength of our study, all hospitalized patients during the established period received the same treatment, without patient selection or management according to the criteria of treating physicians, which allowed making comparisons between patient groups effectively. Another strength was its focus on managing hospitalized patients in general hospital wards, where other studies have not shown any effect of drugs. However, this is also its main limitation since its effect in the intensive care unit was not evaluated. Although a single center is a limitation, it was useful to make the direct comparison between the cohorts by not modifying the conditions of the hospital resources or the hospital staff. The observational design, small population size and single center nature of the study limits the generalizability of the findings.

## Conclusions

In this study, the treatment of severe Covid-19 Pneumonia with high-dose methylprednisolone for three days followed by oral prednisone for 14 days decreased significantly, compared with dexamethasone 6 mg for 7 to 10 days, the recovery time, the need for transfer to intensive care, and the severity markers C-reactive protein (CRP), D-dimer and LDH. Randomized controlled studies with dexamethasone are required to corroborate its effect, and studies in a population hospitalized in intensive care wards.

## Supporting information

S1 Dataset (XLSX)
